# Preface: The Role of Pharmacists in Palliative and End of Life Care

**DOI:** 10.3390/pharmacy9030139

**Published:** 2021-08-17

**Authors:** Eleanor Wilson

**Affiliations:** Nottingham Centre for the Advancement of Research in End of Life Care (NCARE), B302 School of Health Sciences, University of Nottingham, Nottingham NG7 2AH, UK; eleanor.wilson@nottingham.ac.uk

In this Special Issue exploring the role of pharmacists in palliative and end of life care, we sought articles that would shed light on the ways in which pharmacists could impact end of life care. This edition explores issues including the integration of pharmacists in palliative care teams, access to medications, supporting patients in hospital and at home and the types of medications used at the end of life.

Advancements in technologies, pharmaceuticals and health promotion mean that people live longer. Life expectancy in the UK has almost doubled since 1841 [1] and we are now much more likely to die at an older age and after an extended period of living with multiple co-morbidities [2,3]. However, we know little about peoples’ experiences of medication use in the context of palliative and end of life care. There is increasing evidence from research into older age and dementia that illustrates the challenges people face in managing medications [4,5,6]. Regimes can often be complex, involving multiple medications and multiple routes of administration [7,8]. As people approach the end of their lives, some long-term preventative medications may be deprescribed but others, such as those for pain, may be increased. Living longer with more chronic conditions means that in the last year of our lives the majority of care is provided in a home environment, placing increasing demands on family members to provide care and support or undertake the management of medications [9,10]. A small body of literature has begun to highlight the complexities of medication provision and management in the community [10,11,12,13]. A recent study showed that the “work” of managing medications at home can include multiple organisational tasks, as well as understanding and taking medications as prescribed [10]. At the end of life this medicine work sits alongside emotional distress, ongoing practical daily living and the exhaustion that is often intrinsic to caring for someone who is dying.

The current and potential role of pharmacists in palliative care and end of life care is rarely recognised by patients or health professionals [14,15,16,17,18]. The scope for pharmacy, while not without potential barriers, is considerable, especially in light of the COVID-19 pandemic [16,19,20]. The Special Issue aims to move the discussion beyond the potential for pharmacists to be part of palliative care provision by drawing together illustrations of good practice and highlighting the ways in which pharmacists are positively contributing to palliative and end of life care in both the hospital and home environments.
